# Modeling ischemic-type biliary lesion *in vitro* using human expandable intrahepatic cholangiocyte organoids

**DOI:** 10.3389/fbioe.2026.1752804

**Published:** 2026-05-29

**Authors:** Zirong Liu, Liuyang Zhu, Pinsheng Han, Wen Tong, Hao Chi, Sen Liu, Yueyue Yang, Libo Wang, Yamin Zhang, Ze Wang, Long Yang, Yunfeng Cui

**Affiliations:** 1 Tianjin NanKai Hospital, Tianjin Medical University, Tianjin, China; 2 Department of Hepatobiliary Surgery, Tianjin First Central Hospital, Tianjin, China; 3 Nankai University of Medicine College, Tianjin, China; 4 Shandong Cancer Hospital and Institute, Shandong First Medical University and Shandong Academy of Medical Sciences, Shandong, China; 5 First Central Clinical College of Tianjin Medical University, Tianjin, China; 6 Department of Pharmacology, Shenyang Pharmaceutical University, Shenyang, China; 7 College of Life Sciences, Nankai University, Tianjin, China; 8 State Key Laboratory of Druggability Evaluation and Systematic Translational Medicine, Tianjin Institute of Pharmaceutical Research, Tianjin, China; 9 Department of Hepatobiliary and Pancreatic Surgery, Department of Surgery, Tianjin Nankai Hospital, Tianjin, China

**Keywords:** disease model, hypoxia and ischemia, intrahepatic cholangiocyte organoids, ischemic-type biliary lesion, liver transplantation

## Abstract

**Background:**

Ischemic-type biliary lesion (ITBL) remains one of the most common complications following liver transplantation. It is imperative to further explore the occurrence and development mechanism of ITBL. Intrahepatic cholangiocyte organoids (ICOs) derived from human liver tissue replicate the structure and function of bile ducts and serve as an innovative experimental tool for *in vitro* modeling of cholangiopathies.

**Methods:**

In this study, ICOs derived from human liver tissue were cultured under ischemia and hypoxia (IH) conditions to establish an *in vitro* model of ITBL. Immunofluorescence staining, RT-qPCR, Western blotting, and transcriptomic analysis were performed to investigate the effects of IH on ICOs and evaluate the validity of the ITBL model.

**Results:**

IH significantly reduced the diameter and cell viability of ICOs. After exposure to IH for more than 48 h, the proliferation of ICOs decreased, accompanied by increased inflammatory responses and apoptosis. Transcriptomic analysis revealed a landscape of pathophysiological genetic changes in the ITBL model in response to IH.

**Conclusion:**

We have presented a novel ITBL model constructed using expandable human ICOs, which is of great significance for exploring the molecular mechanism and potential therapeutic targets of ITBL.

## Highlights

- Human expandable ICOs exhibited characteristics of cholangiocytes.
- ICOs could replicate the pathological features of ITBL under conditions of ischemia and hypoxia.
- The ITBL model could be used to explore the molecular mechanism and potential therapeutic targets of ITBL.

## 1 Introduction

Ischemic-type biliary lesion (ITBL) denotes a focal or extensive biliary injury resulting from compromised blood supply to the bile ducts (Croome et al., 2022). This condition is prevalent in liver transplantation (LT), transarterial chemotherapy, radiotherapy targeting the hepatic region, cholecystectomy with arterial injury, and certain internal medicine pathologies (Goria et al., 2020). Currently, an increasing number of patients with end-stage liver disease are undergoing LT, yet biliary complications remain a significant cause of transplantation failure and associated mortality, with an incidence ranging from approximately 11% to 35% (Gheorghe et al., 2023). Biliary complications include bile leakage, strictures, and dysfunction and can be categorized as anastomotic or non-anastomotic strictures. ITBL can occur without arterial thrombosis after LT, primarily leading to non-anastomotic stenosis, with an incidence of 1.4%–26% (Jiang et al., 2016). However, effective curative treatments remain largely unavailable for most patients, aside from re-transplantation. Therefore, it is imperative to further explore the occurrence and development mechanism of ITBL and to develop better preventive and therapeutic strategies.

Previous studies have found that ITBL is mainly associated with cold ischemia time and ischemia-reperfusion injury (IRI). It has been elucidated as a sequela of microcirculation dysfunction, hypoxia, oxidative stress, and cell death (Shi et al., 2016; Hofmann et al., 2022). IRI promotes the production of reactive oxygen species in Kupffer cells, which, in turn, facilitates the activation of pro-inflammatory signaling cascades, including TNF-α, IL-6, and interferon, along with the production of damage-associated molecular patterns (Kaltenmeier et al., 2022). However, the specific molecular mechanism by which IRI leads to ITBL remains unclear. Therefore, simulating ischemic and hypoxic injury to cholangiocytes *in vitro* is important for elucidating the molecular mechanisms underlying ITBL and identifying potential therapeutic targets.

Two-dimensional cell lines and rodent models are widely used to develop ITBL models, leading to the discovery of various therapeutic targets and drugs (Bai et al., 2022; Yasen et al., 2023). For instance, one study investigated the role and mechanism of deoxycholic acid in mediating bile duct injury via the FXR-mitochondrial apoptotic pathway using *in vitro* cell stimulation and *in vivo* bile acid-fed mouse models (Wang L. et al., 2024). However, genetically and functionally altered cell lines may compromise the authenticity of the models, and two-dimensional cultures fail to replicate the complexity of the *in vivo* three-dimensional environment. Additionally, animal models often do not accurately represent human diseases due to interspecies differences (Sato et al., 2021). The emergence of human organoids has therefore received widespread attention as a promising approach to overcome these limitations (Kim et al., 2020).

In recent years, rapid advances in organoid technology have facilitated the successful construction of cholangiocyte organoids using induced pluripotent stem cells (iPSCs) and adult stem cells (ASCs), making this a frontier technology for biliary disease research (Sampaziotis et al., 2015; Shiot et al., 2021). Intrahepatic cholangiocyte organoids (ICOs) derived from human liver tissue can replicate the *in vivo* structure and function of bile ducts while also maintaining genetic stability during large-scale culture and extended periods of expansion (Sampaziotis et al., 2021). As an innovative experimental tool for *in vitro* modeling of cholangiopathies, ICOs have been employed to identify toxic drugs that affect the bile ducts (Wang Z. et al., 2024). Shi et al. (2022) successfully recapitulated cholangiopathy-associated necroptosis using ICOs, thereby providing a valuable *in vitro* platform for investigating biliary cytotoxicity and conducting preclinical drug evaluations. Lim et al. (2023) demonstrated that ICOs can support hepatitis B virus infection and replication, suggesting their potential utility in the development of personalized antiviral drug-testing platforms. Furthermore, ICOs derived from patients revealed delayed epithelial development and impaired barrier function in the biliary atresia (Amarachintha et al., 2022; Yao et al., 2023). Some studies also found that human ICO cultures were feasible to simulate biliary ischemia and reoxygenation injury *in vitro*, providing a useful tool for pathogenesis investigation and drug screening of cholangiopathies (Shi et al., 2023; Kreiner et al., 2024). Therefore, ICOs hold promise as a medicinal platform for exploring the molecular mechanisms and therapeutic targets of ITBL.

In this study, the expandable ICOs were generated from normal liver tissue and cultured under conditions of ischemia and hypoxia (IH) to simulate ITBL *in vitro*. The model could replicate key characteristics of ITBL, including hypoxia, inflammatory response, and apoptosis, after 48 h of treatment under IH conditions. The findings demonstrated that the ITBL model derived from ICOs has potential as a novel platform for elucidating the molecular mechanisms of ITBL and assessing therapeutic responses to pharmacological interventions.

## 2 Materials and methods

### 2.1 Human ICO initiation and expansion

Liver tissue specimens (n = 5) were collected from patients undergoing partial hepatectomy due to trauma at Tianjin First Central Hospital and were confirmed as normal liver tissue by pathological examination. The use of human specimens in this study was approved by the Ethics Committee of Tianjin First Central Hospital (2020N221KY). All patients provided informed consent for specimen use. The human ICOs were initiated and expanded as previously described (Sampaziotis et al., 2021). In brief, the tissue was washed twice with PBS, cut into pieces of 0.5–2 mm^3^, washed again with PBS, and allowed to settle naturally. After removing the supernatant, collagenase IV (Solarbio) was added, and the tissue was placed on a shaker at 37 °C for 30 min of digestion. The single-cell suspension was collected by filtration through a 70 μm filter and centrifuged at 300 *g* for 5 min at 4 °C. After cell counting, the cell pellet was resuspended in cold BME (Basement Membrane Extract, Type 2, R&D Systems) and quickly seeded into multi-well plates. Organoid initiation medium (OIM) was added after the BME had solidified, and the cells were incubated at 37 °C with 5% CO_2._ The OIM consisted of advanced DMEM/F12 medium (Gibco) supplemented with 1% penicillin–streptomycin (Gibco), 10 mM HEPES (Gibco), 1% GlutaMax (Gibco), 2% (v/v) B27 supplement (Gibco), 1% N2 supplement (Gibco), 1.25 mM N-acetyl-L-cysteine (MedChemExpress), 10 mM nicotinamide (MedChemExpress), 10 nM gastrin I (MedChemExpress), 10 μM forskolin (MedChemExpress), 5 μM A83-01 (MedChemExpress), 10 μM Y-27632 (Stemcell), 50 ng/mL EGF (Novoprotein), 100 ng/mL FGF10 (Novoprotein), 25 ng/mL HGF (Novoprotein), 25 ng/mL noggin (MedChemExpress), 500 ng/mL Wnt3a (MedChemExpress), and 200 ng/ml R-spondin-1 (MedChemExpress). The OIM was changed every 3 days, and ICOs formed within 5–7 days. When the organoids reached a diameter of 300–500 μm after 10–14 days, they could be mechanically passaged. After passaging, the medium was switched to organoid growth medium (OGM) and refreshed every 3 days. The OGM consisted of advanced DMEM/F12 medium (Gibco) supplemented with 1% penicillin-streptomycin (Gibco), 10 mM HEPES (Gibco), 1% GlutaMax (Gibco), 2% (v/v) B27 supplement, 1% N2 supplement (Invitrogen), 1.25 mM N-acetyl-L-cysteine, 10 mM nicotinamide, 10 nM gastrin I, 10 μM forskolin, 5 μM A83-01, 50 ng/mL EGF, 100 ng/mL FGF10, 25 ng/mL HGF, and 25 ng/mL noggin. After three passages of expansion, the ICOs were utilized for subsequent experiments or cryopreserved.

### 2.2 IH treatment of ICOs

ICOs were cultured in a hypoxia incubator chamber (Precision Biomedicals Co., Ltd.) to simulate ischemia and hypoxia injury of the bile duct *in vitro* (1% O_2_, 5% CO_2_), using advanced DMEM/F12 medium supplemented with 1% penicillin-streptomycin. To explore the effects of different hypoxia and ischemia durations on ICOs, four groups were set up: Control (Con), 24 h, 48 h, and 72 h. Bright-field images were captured using an Olympus microscope, and ImageJ software was employed to analyze changes in organoid diameter.

### 2.3 Cell viability assay

ICOs from different donors were evenly seeded into 96-well assay plates (Corning), and cell viability was detected after IH treatment by a CellTiter-Glo 3D Cell Viability Assay according to the manufacturer’s instructions. Briefly, the culture medium was removed from the plate, and 100 μL of fresh advanced DMEM/F12 medium mixed with an equal volume of CellTiter-Glo® 3D Reagent was added. The contents were mixed vigorously for 5 min to induce cell lysis, followed by incubation at room temperature for an additional 25 min to stabilize the luminescent signal. Luminescence was measured using an enzyme-labeled apparatus (Thermo Fisher Scientific), and the relative cell viability was calculated as the ratio of viability in the IH group to that in the control group (% of control).

### 2.4 RNA isolation and RT-qPCR

ICOs were collected, and total RNA was extracted using the TransZol Up Plus RNA Kit (TransGen Biotech) following the manufacturer’s instructions. RNA concentration and purity were measured using spectrophotometry (Thermo Fisher Scientific). The cDNA synthesis was performed with 5 μg of total RNA per sample using a Transcriptor First Strand cDNA Synthesis Kit (Roche). RT-qPCR was performed by FastStart Universal SYBR Green Master (Roche) according to the manufacturer’s instructions. The primers are listed in Supplementary Table S1, and relative gene expression levels were normalized to GAPDH expression.

### 2.5 Hematoxylin and eosin staining and immunofluorescence

ICOs were collected and fixed with 4% paraformaldehyde (Solarbio) at 4 °C for 12 h, then resuspended in 3% agarose and solidified on ice. After being dehydrated and embedded in paraffin, the ICOs were sectioned into 3-μm-thick sections. For H&E staining, the sections were deparaffinized, rehydrated, and stained using a Hematoxylin-Eosin Stain Kit (Solarbio). For immunofluorescence, antigen retrieval was performed with Tris–EDTA after dewaxing and rehydration. Then, the sections were blocked with 5% BSA for 1 h and permeabilized with 0.1% Triton X-100 for 10 min, followed by incubation with primary antibodies overnight at 4 °C. After washing with PBS, the sections were incubated with secondary antibodies for 2 h at room temperature and washed again. A mounting medium containing DAPI was used to counterstain the cell nucleus, and images were acquired using a total internal reflection fluorescent microscope (TIRF & Thunder, Leica). Antibody details are listed in Supplementary Table S2.

### 2.6 Fluorescein TUNEL staining

Apoptosis levels in ICOs were detected using a TUNEL *In Situ* Apoptosis Kit (Elabscience). In brief, after dewaxing and rehydration, ICO sections were treated with protease K at 37 °C for 15 min, then incubated with TUNEL-FITC at 37 °C for 60 min. A mounting medium containing DAPI was used to counterstain the cell nucleus, and images were acquired by a total internal reflection fluorescent microscope (TIRF & Thunder, Leica).

### 2.7 Western blotting

The total protein from ICOs was extracted using a mixture of radioimmunoprecipitation assay buffer (RIPA) and phenylmethylsulfonyl fluoride (PMSF) (both from Solarbio). Protein quantification was performed using the BCA Protein Assay Kit (GlpBio). Protein samples (40 μg/lane) were separated by 5% or 10% SDS-PAGE (Solarbio) and then transferred to polyvinylidene fluoride (PVDF) membranes (Millipore). The membranes were blocked with 5% skim milk at room temperature for 1 h, then incubated with primary antibodies overnight at 4 °C. Then the membranes were washed three times with Tris-buffered saline-Tween (TBST) and incubated with the corresponding secondary antibodies. The details of primary and secondary antibodies are listed in Supplementary Table S3. Finally, Super Excellent Chemiluminescent Substrate (ECL, Elabscience) was evenly applied to each strip, and images were acquired using a ChemiDoc MP Imaging System (Bio-Rad) and analyzed with ImageJ 7.0 (Image Software).

### 2.8 RNA-sequencing and transcriptomic analysis

Total RNA was extracted from ICOs of different groups (Con, n = 5; IH 48 h, n = 5) and assessed using the Bioanalyzer 2100 system (Agilent). After library preparation and cluster generation, the samples were sequenced on an Illumina NovaSeq platform (Novogene), generating 150 bp paired-end reads. Raw data in fastq format were first processed through fastp software, and clean reads were obtained for downstream analyses (Chen et al., 2018). The reference genome index was built, and paired-end clean reads were aligned to the reference genome using Hisat2 (v2.0.5). The mapped reads were assembled by StringTie (v1.3.3b), and featureCounts (v1.5.0-p3) was used to count the number of reads mapped to each gene. The FPKM of each gene was calculated based on the gene length and read counts. DESeq2 was used to identify differentially expressed genes (DEGs), with genes having an adjusted *p* < 0.05 and |Log2 (foldchange)| > 1 considered differentially expressed. Gene Ontology (GO) and Kyoto Encyclopedia of Genes and Genomes (KEGG) enrichment analyses of DEGs were implemented by the clusterProfiler R package. Gene set enrichment analysis (GSEA) was performed using the Hallmark gene set with GSEA software (v4.1.0).

### 2.9 Forskolin-induced swelling assay

The forskolin-induced swelling assay was performed to examine the cystic fibrosis transmembrane conductance regulator (CFTR) activity in the organoids. This assay was performed following a previously published protocol (Boj et al., 2017). Briefly, the ICOs underwent different treatments and were imaged 0, 6 h, and 24 h after the addition of forskolin (10 μM; Cat. No. CM00273; Proteintech). To quantify the percentage of swelling, areas of the organoids were measured using ImageJ.

### 2.10 Statistical analysis

All data were analyzed using GraphPad Prism 8.0.2 (GraphPad Software) and R software (v3.6.2). Data were presented as the mean ± SD, and the comparisons between multiple groups were conducted by ANOVA with post-hoc tests. *p <* 0.05 was considered statistically significant, and additional details were described in the figure legends.

## 3 Results

### 3.1 Human expandable ICOs possessed characteristics of cholangiocytes

**FIGURE 1:**
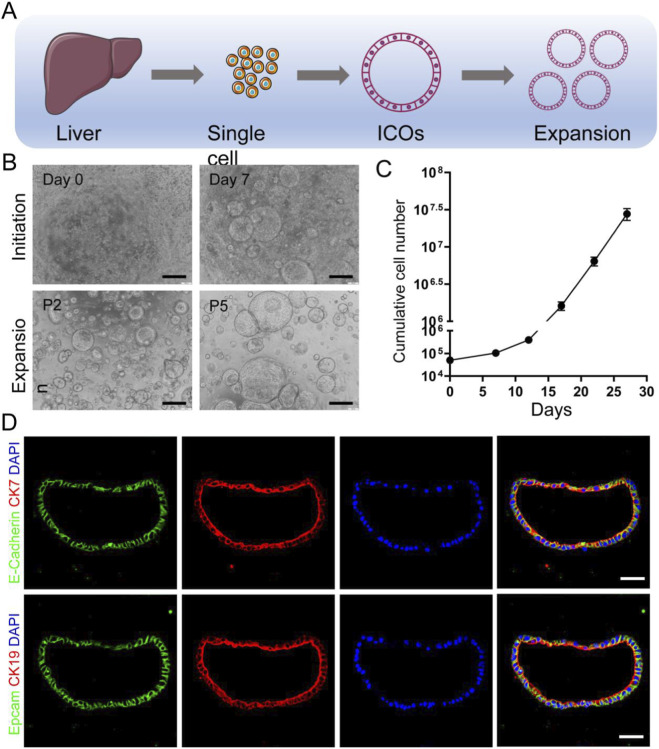
Generation and characterization of human ICOs. **(A)** Schematic diagram of the generation and expansion of human ICOs. **(B)** Representative images of different phases of ICOs, including initiation from day 0 to day 7 and expansion from passage 2 to passage 5. Scale bar, 500 μm. **(C)** Cumulative cell number of ICOs on different days of culture. Data are presented as the mean ± SD. **(D)** Representative immunofluorescence images of ICOs stained with markers of mature cholangiocytes (E-cadherin, CK7, Epcam, and CK19). Scale bar, 50 μm. ICOs, intrahepatic cholangiocyte organoids.

Human ICOs (n = 5) were initiated from liver tissue according to the protocol as previously described (Sampaziotis et al., 2021). Figure 1A shows a schematic diagram of the generation of ICOs. It was observed that ICOs could self-assemble within 5–7 days and form a monolayer cystic structure (Figure 1B). In addition, the induced ICOs could be rapidly passaged and exhibited long-term expansion capacity (Figures 1B,C). Immunofluorescence results showed that Epcam, CK19, E-cadherin, and CK7 were expressed in ICOs (Figure 1D), which were the markers of mature cholangiocytes. Together, the above results demonstrated that the expandable ICOs possessed characteristics of cholangiocytes *in vitro*.

### 3.2 IH affected the morphology and cell viability of ICOs

**FIGURE 2:**
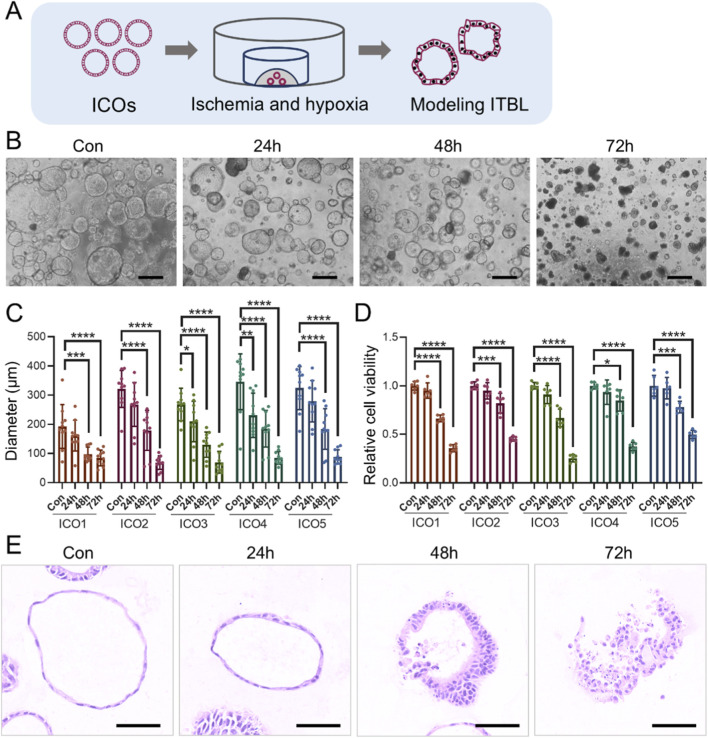
IH affected the growth and cell viability of ICOs. **(A)** Schematic diagram of the ITBL model derived from ICOs. **(B)** Representative images of ICOs under IH for 0 h (Con), 24 h, 48 h, and 72 h. Scale bar, 200 μm. **(C)** The diameter of ICOs (n = 10 per donor, five donors) after different durations of IH treatment was measured and analyzed using ImageJ software. **(D)** Relative cell viability of ICOs (n = 6 per donor, five donors) after different durations of IH treatment, normalized to the control group. **(E)** Histopathological features of ICOs after different durations of IH treatment. Scale bar, 100 μm. Data are presented as mean ± SD. *p < 0.05, **p < 0.01, ***p < 0.001, and ****p < 0.0001; ns, no significant difference. IH, ischemia and hypoxia; ITBL: ischemic-type biliary lesion.

To mimic ITBL, ICOs were cultured in a hypoxia incubator chamber under low-oxygen tension in a serum-free medium (Figure 2A). Different durations (0 h, 24 h, 48 h, and 72 h) were used to explore the effects of IH on ICOs. The results showed that with prolonged IH exposure, the diameter of ICOs decreased and the lumen appeared darker under bright field microscopy (Figure 2B). Statistical analysis revealed that the diameter of ICOs derived from different donors decreased significantly after 48 h and 72 h of IH treatment (Figure 2C). The cell viability of ICOs also decreased significantly after 48 h and 72 h of IH treatment (Figure 2D). In addition, H&E staining results showed that ICOs in the Control group exhibited a flat single-layered epithelium, with no significant morphological changes observed after 24 h of treatment. However, after 48 h, ICOs showed irregular morphological changes, such as rupture of intercellular junctions, while the organoid morphology was markedly disrupted after 72 h (Figure 2E).

### 3.3 IH reduced proliferative capacity and promoted apoptosis of ICOs

**FIGURE 3:**
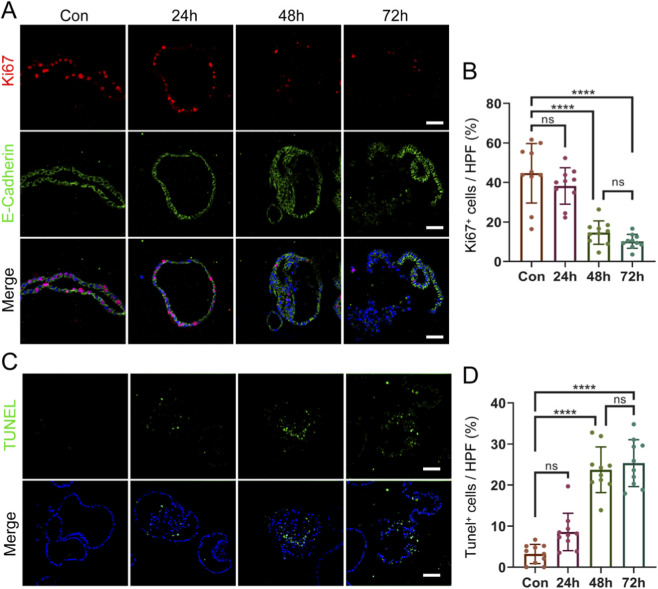
IH affected the proliferation and apoptosis of ICOs. **(A)** Representative immunofluorescence images of ICOs stained with E-cadherin and Ki67 after different durations of IH treatment. Scale bar, 50 μm. **(B)** Quantification of Ki67-positive cells per high-power field (n = 10) in each group, performed using ImageJ software. **(C)** Representative immunofluorescence images of ICOs stained with TUNEL after different durations of IH treatment. Scale bar, 50 μm. **(D)** Quantification of TUNEL-positive cells per high-power field (n = 10) in each group, performed using ImageJ software. Data are presented as mean ± SD. *p < 0.05, **p < 0.01, ***p < 0.001, and ****p < 0.0001; ns, no significant difference.

It had been demonstrated that ICOs possess robust proliferative capacity, similar to stem/progenitor cells around the biliary system *in vivo*. The reduction in diameter and decreased cell viability following IH treatment suggested diminished proliferative capacity of the ICOs. Therefore, immunofluorescence staining for Ki67 was performed, and the results indicated that approximately 40% of cells in ICOs of the Control group expressed Ki67 (Figure 3A). However, the number of Ki67-positive cells significantly decreased after more than 48 h of IH treatment, with no significant difference observed between the 48 h and 72 h groups (Figures 3A,B). In addition, E-cadherin, a transmembrane glycoprotein highly expressed in bile duct epithelium, is essential for maintaining normal morphology and polarity. Immunofluorescence staining showed that the ICOs structures were disrupted after IH, accompanied by decreased E-cadherin expression, particularly at 72 h (Figure 3A). The TUNEL results indicated that the proportion of TUNEL-positive cells increased significantly after more than 48 h of IH treatment (Figures 3C,D). Together, these results indicated that ICOs showed a significant decrease in proliferation and an increase in apoptosis after 48 h of IH treatment.

### 3.4 ITBL model derived from ICOs showed an inflammatory response and apoptosis

**FIGURE 4:**
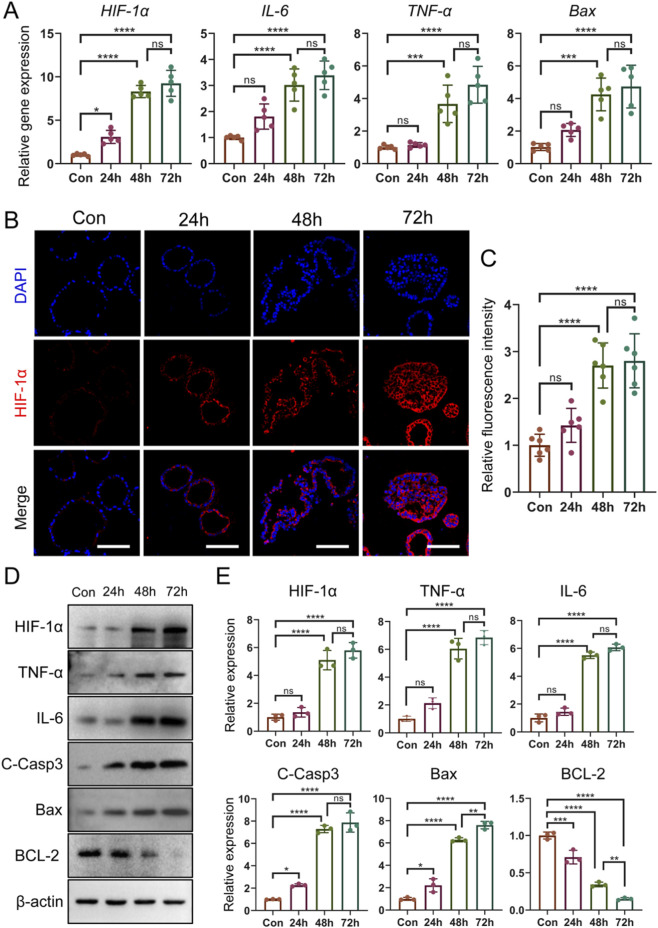
Modeling ITBL using ICOs after 48 h of IH treatment. **(A)** Relative mRNA expression levels (n = 5) of specific genes related to hypoxia, inflammatory response, and apoptosis after different durations of IH treatment. **(B)** Representative immunofluorescence images of ICOs stained with HIF-1α after different durations of IH treatment. Scale bar, 100 μm. **(C)** The relative fluorescence intensity of HIF-1α (n = 6) in the four groups. **(D)** Western blot analysis of protein levels related to hypoxia, inflammatory response, and apoptosis after IH treatment. **(E)** Quantification of protein levels shown in **(D)** with β-actin as the loading control. Data are presented as mean ± SD. *p < 0.05, **p < 0.01, ***p < 0.001, and ****p < 0.0001; ns, no significant difference. C-Casp3, Cleaved-caspase3.

Under IH conditions, the organism initiates compensatory regulatory mechanisms, among which hypoxia-inducible factor (HIF) plays a pivotal role by activating a cascade of inflammatory factors, thereby inducing an inflammatory response and apoptosis (McGettrick and O'Neill, 2020). After IH treatment, the mRNA expression levels of *HIF-1α*, pro-inflammatory factors (*IL-6*, *TNF-α*), and the apoptosis-related gene (*Bax*) in ICOs were significantly increased, but there was no further increase upon treatment for 72 h compared to 48 h (Figure 4A). Immunofluorescence staining showed that HIF-1α was expressed in ICOs after IH, and the relative fluorescence intensity of HIF-1α increased after 48 h (Figure 4B). Consistent with these findings, WB results showed that the protein levels of HIF-1α, pro-inflammatory factors (IL-6, TNF-α), and pro-apoptotic proteins (Bax, C-Caspase3) were significantly increased, while the anti-apoptotic protein (Bcl-2) decreased after 48 h and 72 h of IH treatment (Figure 4C). Furthermore, there were no significant differences in the protein levels of HIF-1α, TNF-α, and C-caspase3 between the 48 h and 72 h groups. These results suggested that ICOs treated with IH for 48 h could replicate the inflammatory response and apoptosis characteristic of ITBL *in vitro*.

### 3.5 Transcriptomic profile revealed characteristics of the ITBL model derived from ICOs

**FIGURE 5:**
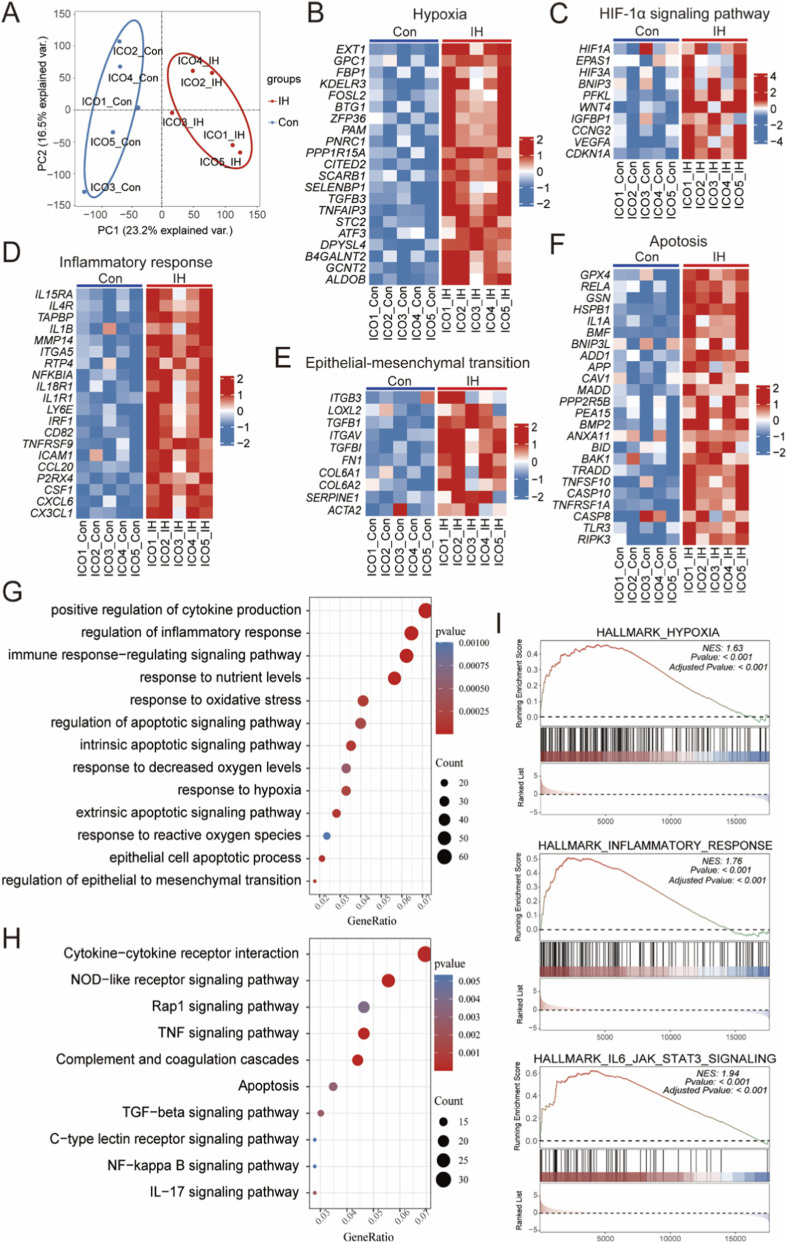
Transcriptomic analysis of the ITBL model constructed using ICOs. **(A)** PCA of 10 samples (Con, n = 5; IH 48h, n = 5) based on gene expression profiles. **(B–F)** Heatmaps showing the expression of genes related to hypoxia, HIF-1α signaling pathway, inflammatory response, EMT, and apoptosis. **(G–H)** GO and KEGG enrichment analysis of DEGs upregulated in the IH group. **(I)** GSEA of hallmark gene set signatures in the IH group. PCA, principal component analysis; EMT, epithelial–mesenchymal transition; DEGs, differentially expressed genes; GO, Gene Ontology; KEGG, Kyoto Encyclopedia of Genes and Genomes; GSEA, gene set enrichment analysis.

To further characterize the overall genomic changes in the constructed ITBL model, transcriptome analysis was performed on ICOs treated with IH for 48 h compared to the Control group. The principal component analysis (PCA) plot showed that the IH and Control groups tended to cluster separately based on common features of gene expression (Figure 5A). The gene expression heatmap revealed a global upregulation of hypoxia-related genes in the IH group, indicating that ICOs responded specifically to hypoxia (Figure 5B). Genes in the HIF-1α signaling pathway, including *HIF1A*, *EPAS1*, and *HIF3A*, were also significantly upregulated (Figure 5C). In addition, genes related to inflammatory response and apoptosis were upregulated (Figures 5D,F). Previous studies have found that epithelial–mesenchymal transition (EMT) may play an important role in biliary fibrosis in ITBL, and the heatmap showed that EMT-related genes (*LOXL2, TGFB1, COL6A1, ACTA2, etc*.) were significantly upregulated (Figure 5E). GO enrichment analysis revealed that the DEGs upregulated in the IH group were mainly involved in the biological processes related to hypoxia, inflammatory response, apoptosis, and EMT (Figure 5G). KEGG enrichment analysis showed that the DEGs were mainly enriched in cytokine–cytokine receptor interaction, TNF signaling pathway, apoptosis, TGF-β signaling pathway, *etc.* (Figure 5H). GSEA of hallmark gene sets showed enrichment for hypoxia, inflammatory response, and IL6_JAK_STAT3_Signaling in the IH group (Figure 5I). Collectively, these results demonstrated that the transcriptome changes in the ITBL model from ICOs were mainly concentrated in hypoxia, inflammatory response, and apoptosis.

### 3.6 IH impairs barrier integrity and function of ICOs in the ITBL model

**FIGURE 6:**
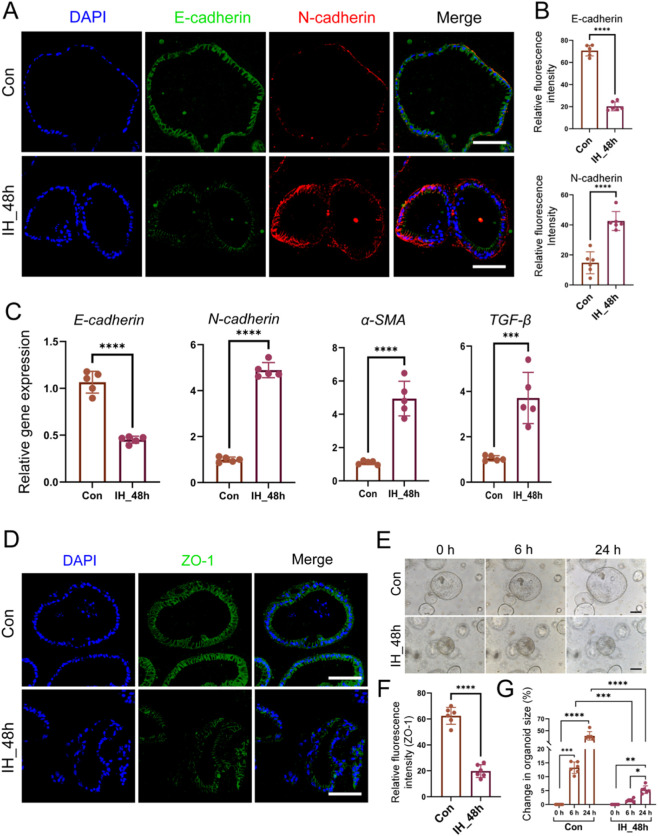
IH affects the barrier function of ICOs. **(A)** Representative immunofluorescence images of ICOs stained with E-cadherin and N-cadherin after IH_48 h. **(B)** Quantification of E-cadherin and N-cadherin positive cells per high-power field (n = 6) in each group, performed using ImageJ software. **(C)** The relative mRNA expression levels (n = 6) of E-cadherin, N-cadherin, α-SMA, and TGF-β after IH_48 h. **(D)** Control and ICOs after IH_48 h were fixed and immunostained for ZO-1. **(E)** The CFTR activity of the Control and ICOs after IH_48 h was assayed using the forskolin-induced swelling assay. **(F)** Fluorescence intensities of ZO-1 in the Control and ICOs after IH_48 h. **(G)** Size of the Control and ICOs after IH treatment for 48 h at 0, 6 h, and 24 h after forskolin (10 µM) treatment.

EMT is one of the mechanisms that contributes to the pathogenesis of liver and biliary fibrosis in different diseases. To validate the activation of EMT-related pathways in the ITBL model, we performed immunofluorescence (Figure 6A). The results revealed that, compared to the control group, the ICOs after IH_48 h exhibited lower E-cadherin expression, but N-cadherin expression was significantly upregulated (Figure 6B). Further RT-qPCR validated the RNA-seq results for *CDH1* and *CDH2* (Figure 6C). Genes involved in fibrosis (*α-SMA* and *TGF-β*) were found to be upregulated after IH for 48 h (Figure 6C). Upregulation of N-cadherin (encoded by CDH2) and downregulation or unchanged expression of E-cadherin (CDH1) are indicators of EMT (Loh et al., 2019; Yang et al., 2020).

EMT could drive the fibrosis observed in cholangiopathy, which may explain the morphological changes observed in the ITBL model derived from ICOs. Thus, we next sought to examine whether the function of the ITBL model derived from ICOs was affected by low oxygen tension and serum-free medium. We first performed the forskolin-induced swelling assay, which measures the activity of cystic fibrosis transmembrane conductance regulator (CFTR) in cholangiocytes (Figure 6E) (Ogawa et al., 2021; Verstegen et al., 2020). Although the size of the control organoids increased by ∼50% following forskolin treatment, the size of the ICOs after IH_48 h remained similar to that of the untreated ICOs (Figure 6G).

Because EMT can lead to the breakdown of epithelial tight junctions (Yang et al., 2020), we then investigated whether IH_48 h could lead to the downregulation of the tight junction-related protein ZO-1 on endothelial and various epithelial cells (Tian et al., 2018; Tugizov et al., 1996; Maidji et al., 1996). In the ICOs after IH_48 h, there was a decreased fluorescence intensity for ZO-1 compared to the control ICOs (Figures 6D,F). Together, these data show that IH induced substantial changes in the barrier integrity and function of the ICOs in the ITBL model.

## 4 Discussion

ITBL has consistently been one of the most common complications after LT and significantly reduces the survival rates of patients and grafts. ITBL causes atypical inflammatory reactions and intrahepatic cholestasis, leading to bile duct proliferation, fibrosis, or necrosis (Dobrindt et al., 2020). Previous studies have implicated cold ischemia time, a hepatic phase, and immunological rejection as key risk factors for ITBL, but the precise mechanism underlying its occurrence and progression remains obscure (Hessheimer et al., 2016; Durán et al., 2023). Hemodynamics and oxygen supply are presumed to play significant roles in ITBL development. Hence, in this study, we employed ICOs to simulate the pathologic characteristics of ITBL under IH conditions *in vitro*. The model also holds certain value for mimicking IH injury of the graft in organ transplantation.

Unlike IRI, ITBL is characterized by ongoing, non-resolving biliary epithelial damage (Durán et al., 2023). The underlying pathology involves microcirculatory dysfunction of the peribiliary vascular plexus rather than compromise of the main hepatic artery, resulting in sustained hypoxic injury to cholangiocytes. The model developed by Shi et al. (2023) focused on simulating the acute phase of IRI, specifically the response of cholangiocytes to reoxygenation after hypoxic exposure. In contrast, our ITBL model was designed to recapitulate the persistent, non-reversible hypoxic injury that characterizes the post-transplant clinical course of ITBL. In our model, ICOs were subjected to continuous ischemia and hypoxia without reoxygenation, leading to sustained-inflammatory responses, activation of apoptotic pathways, and impaired barrier function. These features are not captured in ischemia-reoxygenation models.

Over the past decades, clinical research has significantly expanded our understanding of ITBL. However, the detailed molecular mechanism of ITBL remains largely unexplored due to the lack of suitable models, thereby hindering the development of therapeutic strategies. Zhao et al. established an animal model of ischemic bile duct stricture in mice and observed significant ITBL of the extrahepatic bile duct 21 days after surgery (Zhao et al., 2008). Sheng et al. (2009) also established a rabbit model of ischemic-type intrahepatic biliary lesion with blood deficiency by clamping the hepatic artery and common bile duct. Compared with cell lines and animal models, ICOs derived from human liver tissue more closely resemble the histological and genetic features of the *in vivo* environment (Roos et al., 2021). We previously reported that human liver organoids were used to establish an ischemia-reperfusion injury model for investigating the therapeutic efficacy of TWEAK (Tong et al., 2024). ICOs also have multiple advantages in disease modeling and drug response testing, owing to their capability to simulate intercellular communication, replicate organ structures, and maintain cells’ original phenotypes (Babboni et al., 2024). Many studies have utilized ICOs to construct models, including drug-induced bile duct injury, primary sclerosing cholangitis, and biliary atresia (Shiot et al., 2021). According to previously established methods, expandable ICOs were induced and cultured in serum-free medium under hypoxic conditions to mimic ITBL (Shi et al., 2023). It was obvious that ICOs showed decreased diameter and cell viability after more than 48 h of IH treatment, accompanied by reduced proliferation and increased apoptosis.

As is well known, hypoxia and inflammation frequently coexist and may exacerbate one another through multiple, bidirectional links between molecular pathways (McGettrick and O'Neill, 2020). As a central regulatory factor in the cellular oxygen homeostasis repair mechanism, HIF-1α modulates processes such as cell growth, proliferation, migration, inflammation, and apoptosis (Watts and Walmsley, 2019). HIF-1α expression is often upregulated during inflammation. By regulating the expression of related target genes such as vascular endothelial growth factor (VEGF) and prostaglandin E2 (PGE2), HIF-1α induces endothelial cell dysfunction and angiogenesis, thereby promoting inflammation and apoptosis (Luo et al., 2022). This study found that the expressions of hypoxia, inflammation, and apoptosis-related genes were significantly upregulated after more than 48 h of IH treatment, but there was no significant difference between 72 h and 48 h treatments. Consistent with the above results, protein quantification also indicated that ICOs could recapitulate the pathological response of ITBL *in vitro* after 48 h of IH treatment. Therefore, in this ITBL model derived from ICOs under IH conditions, the expression of HIF-1α was upregulated, which further promoted inflammation and apoptosis in organoids.

Transcriptomic analysis further revealed the gene expression changes in the constructed ITBL model. GO and GSEA enrichment analyses showed significant enrichment of hypoxia-related pathways in ICOs after IH treatment, and hypoxia-related genes were also upregulated, representing a typical response to IH. There was a general upregulation of HIF family genes (*HNF1A*, *EPAS1*, and *HNF3A*) and HIF-target genes, such as *BNIP3*, *PFKL*, *WNT4*, and *CDKN1A*. In addition, analysis of gene expression features of several signaling pathways showed the mechanism of bile duct injury in ITBL induced by IH. For instance, inflammation and apoptosis-related pathways were most obviously enriched after IH treatment, including the TNF signaling pathway, the IL6_JAK_STAT3 signaling pathway, and the intrinsic and extrinsic apoptotic signaling pathway (Vanamee and Faustman, 2023). These results also suggested that IH could upregulate HIF gene expression in ICOs, thereby activating inflammation and apoptosis-related pathways and inhibiting cell proliferation. Moreover, upregulated genes in the model were also enriched in the EMT pathway, which is a key factor in ITBL, primary sclerosing cholangitis, and biliary atresia (Wickramaratne et al., 2021). Our results also demonstrate that the IH process upregulates the expression of N-cadherin, α-SMA, and TGF-β associated with EMT and fibrosis and severely affects the barrier function of ICOs.

As a formidable technology, significant advances in the ICOs allow us to better understand the molecular pathways of cholangiopathies. The ITBL model constructed in this study further demonstrated the strong application potential of ICOs, although certain limitations remain. First, ICOs lack immune cells and stromal cells, which play important roles in the development of ITBL. Co-culture with other cell types would be preferable to reflect the tissue complexity and heterotypic cell-cell interactions (Babboni et al., 2024). In addition, the organoid differentiation conditions must be further optimized and standardized to achieve characteristics more closely resembling those *in vivo*. Matrigel remains the commonly used matrix for supporting organoid growth, but it may introduce xenogenic contaminants or induce immune responses, potentially affecting the stability of the ITBL model (Gan et al., 2023). Therefore, more clinically relevant matrices that possess the biological and mechanical properties of the native extracellular matrix (ECM) are needed; synthetic matrix and decellularized ECM may be better options. Finally, with the update and iteration of organoid technology, the three-dimensional research system of vascularized organoids and organoid chips can better simulate the *in vivo* environment and more accurately explore reactive oxygen species bursts, endothelial barrier damage, changes in vascular secretion factors, and more in the study of ischemia and hypoxia (Zhao et al., 2021). In the future, the use of vascularized organoids or organoid chips for ITBL research will be the exploration direction of our team.

## 5 Conclusion

In this study, we successfully constructed an ITBL model using expandable human ICOs. Under IH conditions for 48 h, the model recapitulated key pathological features of ITBL, including decreased proliferation, inflammatory response, and apoptosis, accompanied by upregulation of HIF-1α and activation of related signaling pathways. Transcriptomic analysis revealed enrichment of hypoxia, inflammation, apoptosis, and EMT pathways. Further research data show that ITBL induced substantial changes in the barrier integrity and function of the ICOs. This ICO-based ITBL model provides a valuable platform for investigating the molecular mechanisms of ITBL and exploring potential therapeutic targets, as well as for evaluating drug interventions.

## Data Availability

The datasets presented in this study can be found in online repositories. The names of the repository/repositories and accession number(s) can be found at https://www.ncbi.nlm.nih.gov/, PRJNA1127678.
