# Supplementary material for: Modeling ischemic-type biliary lesion in vitro using human expandable intrahepatic cholangiocyte organoids
Source: Front Bioeng Biotechnol. 2026 May 29;14:1752804. doi: 10.3389/fbioe.2026.1752804 (PMC13260613; doi:10.3389/fbioe.2026.1752804)
Supplement: Supplementary file 1 [file Table1.docx]

| **Supplementary Table 1: List of the primer of RT-qPCR performed in the experiments.** | | |
| --- | --- | --- |
| Gene | Forward (5'-3') | Reverse (5'-3') |
| *HIF-1α* | GAACGTCGAAAAGAAAAGTCTCG | CCTTATCAAGATGCGAACTCACA |
| *TNF-α* | GCTCCAGGCGGTGCTTGTTC | CCAGAGGGCTGATTAGAGAGAGGTC |
| *IL-6* | CACTGGTCTTTTGGAGTTTGAG | GGACTTTTGTACTCATCTGCAC |
| *Bax* | CCCGAGAGGTCTTTTTCCGAG | CCAGCCCATGATGGTTCTGAT |
| *E-cadherin* | CGAGAGCTACACGTTCACGG | GGGTGTCGAGGGAAAAATAGG |
| *N-cadherin* | TGCGGTACAGTGTAACTGGG | GAAACCGGGCTATCTGCTCG |
| *α-SMA* | GTGTTGCCCCTGAAGAGCAT | GCTGGGACATTGAAAGTCTCA |
| *TGF-β* | GGCCAGATCCTGTCCAAGC | GTGGGTTTCCACCATTAGCAC |
| *GAPDH* | GGAGCGAGATCCCTCCAAAAT | GGCTGTTGTCATACTTCTCATGG |

| **Supplementary Table 2. List of the first and secondary antibodies for immunofluorescence in the experiments.** | | | | |
| --- | --- | --- | --- | --- |
| Antibodies | Source | Host | Identifier | Dilution |
| EpCAM | Proteintech | Mouse | 66316-1-Ig | 1:200 |
| E-Cadherin | Proteintech | Mouse | 60335-1-Ig | 1:200 |
| N-Cadherin | Proteintech | Mouse | 66219-1-Ig | 1:200 |
| α-SMA | Proteintech | Mouse | 67735-1-Ig | 1:200 |
| TGF-β | Proteintech | Mouse | 81746-2-RR | 1:200 |
| ZO-1 | Proteintech | Mouse | 66452-1-Ig | 1:200 |
| CK19 | Proteintech | Rabbit | 10712-1-AP | 1:200 |
| CK7 | Proteintech | Rabbit | 17513-1-AP | 1:200 |
| HIF-1α | Proteintech | Rabbit | 20960-1-AP | 1:200 |
| Ki67 | Proteintech | Rabbit | 27309-1-AP | 1:400 |
| Goat Anti-Mouse IgG H&L (Alexa Fluor® 488) | Abcam | Goat | ab150113 | 1:200 |
| Goat Anti-Rabbit IgG H&L (Alexa Fluor® 594) | Abcam | Goat | Ab150080 | 1:200 |

| **Supplementary Table 3. List of the first and secondary antibodies for WB in the experiments.** | | | | |
| --- | --- | --- | --- | --- |
| Antibodies | Source | Host | Identifier | Dilution |
| HIF-1α | Proteintech | Rabbit | 20960-1-AP | 1:3000 |
| TNF-α | Proteintech | Rabbit | 17590-1-AP | 1:1000 |
| IL-6 | Proteintech | Rabbit | 21865-1-AP | 1:1000 |
| Caspase3 | Proteintech | Rabbit | 19677-1-AP | 1:1000 |
| Bax | Proteintech | Rabbit | 50599-2-Ig | 1:2000 |
| Bcl-2 | Proteintech | Rabbit | 12789-1-AP | 1:3000 |
| β-actin | Proteintech | Rabbit | 81115-1-RR | 1:5000 |
| HRP Conjugated AffiniPure Goat Anti-rabbit lgG (H+L) | Boster | Rabbit | BA1055 | 1:1000 |
